# Unveiling Gas Evolution in Sustainable Aqueous Batteries by Online Electrochemical Mass Spectrometry: Progress and Perspectives

**DOI:** 10.1021/acsmaterialsau.5c00124

**Published:** 2025-09-30

**Authors:** Leiting Zhang

**Affiliations:** Department of Chemistry−Ångström Laboratory, 8097 Uppsala University, Box 538, 751 20 Uppsala, Sweden

**Keywords:** online electrochemical mass spectrometry, aqueous batteries, operando gas analysis, hydrogen evolution reaction, oxygen evolution reaction

## Abstract

Aqueous batteries are sustainable energy storage solutions for next-generation grid energy storage. However, their practical deployment is limited by the narrow electrochemical stability window of water, which constrains cell voltage and leads to persistent performance degradation. In this Perspective, online electrochemical mass spectrometry is highlighted as a powerful *operando* technique for detecting and quantifying gas evolution in aqueous batteries. The fundamental principle and historical development of the technique are briefly reviewed, followed by a systematic evaluation of recent advances in applying the technique to study common gassing events in aqueous chemistries. Perspectives on leveraging the technique for high-sensitivity, high-accuracy, and high-throughput investigations of key cell components are offered, with the goal of accelerating the development of robust and commercially viable aqueous batteries.

## Graphical abstract

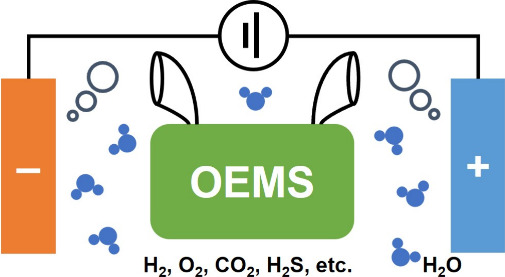

## 1 Introduction

The urgent need to mitigate environmental impacts while meeting growing global energy demands has accelerated interest in sustainable energy storage technologies, with aqueous batteries emerging as a promising candidate. Compared to conventional nonaqueous Li-ion batteries (LiBs), aqueous batteries offer several compelling advantages, evidenced by their intrinsic nonflammability, environmental benignity, and reduced reliance on critical strategic raw materials (Co, Ni, graphite, etc.). These features make aqueous batteries particularly attractive for next-generation large-scale energy storage systems, a trillion-dollar market that remains largely underdeveloped.

The origins of aqueous batteries can be traced back to the early 19th century, when Alessandro Volta demonstrated primary energy storage using alternating layers of Zn and Cu foils, separated by brine-soaked cloth. The first rechargeable aqueous batteries were later invented by Gaston Planté and optimized by Camille Fauré, employing Pb-based electrodes and sulfuric acid–based electrolytes. Over time, a variety of Ni-based aqueous chemistries were developed, including Ni–Cd and Ni-MH batteries, some of which can still be found in commercial products, such as uninterrupted power supply (UPS), consumer electronics, and hybrid vehicles.

Despite these historical advancements, a key limitation of aqueous batteries lies in the narrow electrochemical stability window (ESW) of water (1.23 V).^1,2^ Exceeding this window triggers water electrolysis, resulting in parasitic oxygen evolution reaction (OER) and hydrogen evolution reaction (HER), lowering the Coulombic efficiency and energy efficiency. In particular, the onset of HER is pH-dependent, governed by E_HER_ = −0.059 × pH. At neutral pH, the HER potential is – 0.413 V vs standard hydrogen electrode (SHE), corresponding to 2.627 V vs Li^+^/Li^0^. This potential is more negative than that of nearly all conventional negative electrode materials in LiBs, suggesting that aqueous batteries are inherently prone to cathodic instability. Metal anodes, such as Zn, Fe, Mn, and Al, all suffer from this challenge.

In the past decade, numerous thermodynamic and kinetic strategies have been explored to expand the water ESW, such as using highly concentrated electrolytes, developing electrolyte cosolvents/additives, and applying electrode coatings.^3–10^ Electrochemical techniques like linear sweep voltammetry (LSV) are commonly employed to assess the delayed onset of HER.^11^ However, inconsistencies in the definition of “onset potential” across studies hinder direct comparisons. Moreover, in the presence of active electrode materials, the measured current is a combination of ion insertion and HER, which cannot be easily decoupled.

A straightforward approach to evaluating electrolyte stability is by analyzing gaseous products formed inside aqueous batteries, the central theme of this Perspective. Here, online electrochemical mass spectrometry (OEMS) is highlighted as a powerful *operando* technique for aqueous batteries. The principles and historical development of OEMS are briefly introduced, followed by selected case studies that illustrate its efficacy. Finally, perspectives are presented to promote further utilization of the technique within the expanding aqueous battery community. For applications of the technique in nonaqueous battery chemistries, readers are referred to recent comprehensive review papers.^12–14^

## 2 Online Electrochemical Mass Spectrometry (OEMS)

The OEMS technique, sometimes referred to as DEMS (differential electrochemical mass spectrometry) and EC-MS (electrochemical mass spectrometry), uses a commercial mass spectrometer to quantify the (electro)­chemically developed gases in an electrochemical system in real-time. It is worth mentioning that there is a subtle difference between OEMS and DEMS – a differential pumping system is required for the latter. However, the two abbreviations are often used interchangeably and will not be differentiated herein.

The three essential components of an OEMS system are an electrochemical cell, a mass spectrometer (MS), and an interface connecting the two. Depending on the gas-sampling mechanisms, two configurations are commonly utilized and illustrated in Figure 1. The (a) membrane-inlet approach refers to the system where electrode materials are typically coated on a Teflon membrane, which is in direct contact with the high vacuum of an MS through a glass frit. Such a configuration is primarily designed for aqueous electrocatalysis applications, where fast (subsecond) response time can be realized.

**Figure 1:**
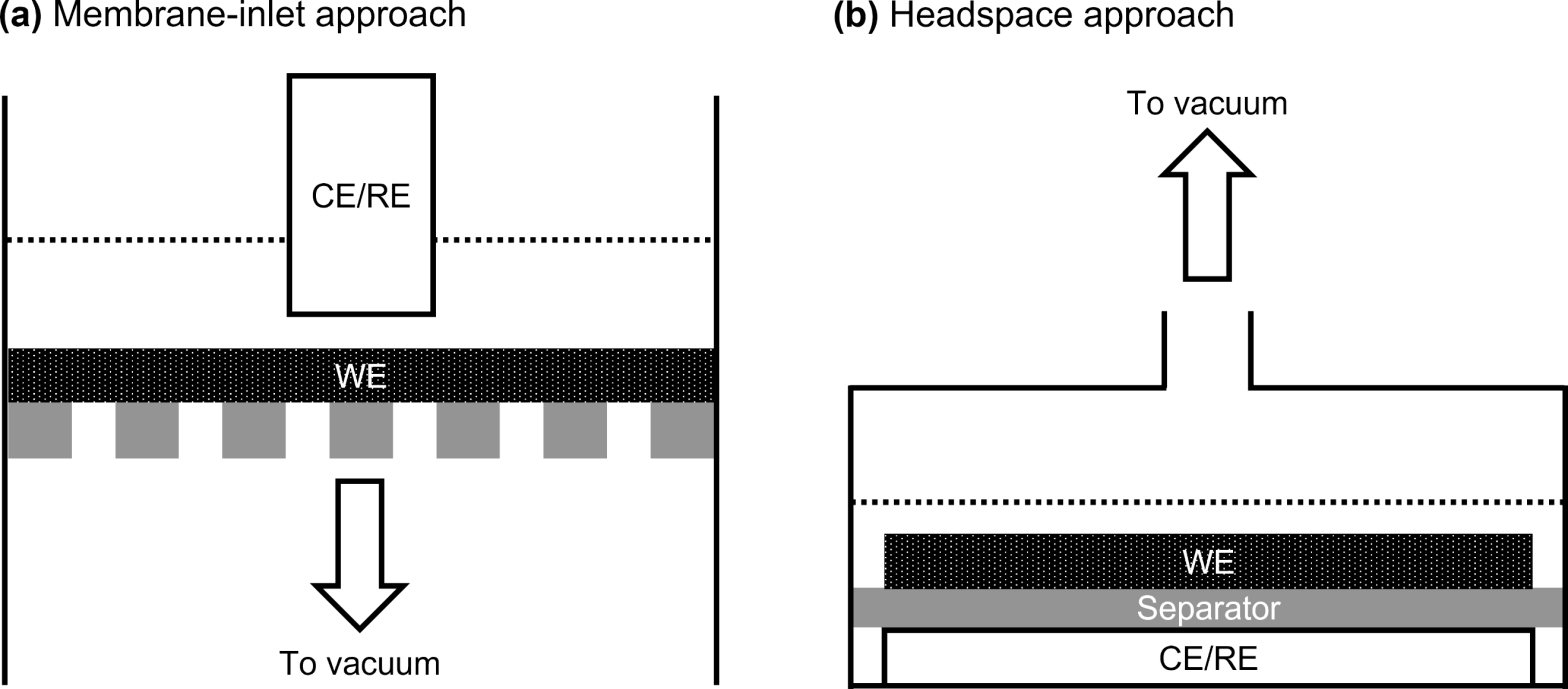
Schematic diagram of OEMS cells following (a) membrane-inlet approach and (b) headspace approach.

An alternative (b) headspace approach was proposed by Novák et al.^15^ and recently improved by Berg et al.^16^ to study gas evolution in batteries. Gases accumulated in the headspace of the battery cell are either continuously or intermittently introduced to the MS through a capillary. Although a slightly longer response time is required, this design minimizes the electrolyte contamination of the MS, notably improving the signal-to-noise ratios of gaseous products. Therefore, most conventional OEMS systems for batteries are designed based on the headspace approach.^17–20^

The quantification of gas evolution can be achieved as follows. The ion current of gas *j* (*I*_*j*_) can be correlated with its partial pressure (*P*_*j*_), following *I*_*j*_ = *S*_*j*_ *P*_*j*_ + *B*_*j*_, where *S*_*j*_ is the machine-specific sensitivity factor and *B*_*j*_ is the background factor. Both *S*_*j*_ and *B*_*j*_ are obtained using a calibration gas with a known concentration of gas *j*. The partial pressure thus obtained can be used to solve the molar amount of *j* (*n*_*j*_), following the ideal gas law: *P*_*j*_ *V* = n_*j*_ *RT*, where V is the gas volume, R is the universal gas constant, and T is the temperature. For a detailed mathematical derivation, readers are advised to refer to ref 16, which extensively discusses the design and validation of an OEMS system based on the headspace approach.

## 3 Representative Gases Stemming from Aqueous Batteries

### 3.1 H_2_

As hydrogen evolution is the most notorious side reaction for aqueous batteries, the OEMS technique has been frequently implemented to identify the onset and extent of HER, or to prove the efficacy of the respective interfacial passivation strategy.^21–25^ Recently, Yik and co-workers designed a robot-assisted high-throughput electrolyte formulation, coin cell assembly, and electrochemical testing platform, ODACell 2, to screen aqueous electrolyte compositions with two salts (LiClO_4_ and LiTFSI) and four solvents (dimethyl sulfoxide, trimethyl phosphate, acetonitrile, and water).^26^

The optimized electrolyte offered 96% Coulombic efficiency and 165 mAh g^–1^ reversible capacity (normalized to LTO) for a LiFePO_4_ | 1 m LiX | Li_4_Ti_5_O_12_ model chemistry (X denotes electrolyte salt anion). To rationalize the improvement, the authors conducted OEMS tests on several electrolyte formulations proposed by Bayesian optimization. As illustrated in Figure 2(a), the authors observed a log–log correlation between the water molar percentage in the electrolyte and the average H_2_ evolution rate. Most electrolyte formulations identified by Bayesian optimization lie on the line, suggesting that reducing the electrolyte water content, ranging from 100% to 14%, decreases the HER rate. However, no synergistic correlation was observed among the cosolvents, as no points fell below the log–log line. Instead, the authors found a peculiar electrolyte composition of 80:20 mol % ACN/H_2_O, which produced significantly more H_2_ than predicted along the trend line. With OEMS, it turned out to be a competing electrochemical reduction of acetonitrile, resulting in the formation of ethylamine (Figure 2(b)).^27^ Such a result is in agreement with a recent study by Paillot and co-workers, who quantified hydrogen evolution from aqueous magnesium batteries by gas chromatography (GC), and concluded that HER was predominantly determined by the system’s water content but not electrolyte structure.^28^

**Figure 2:**
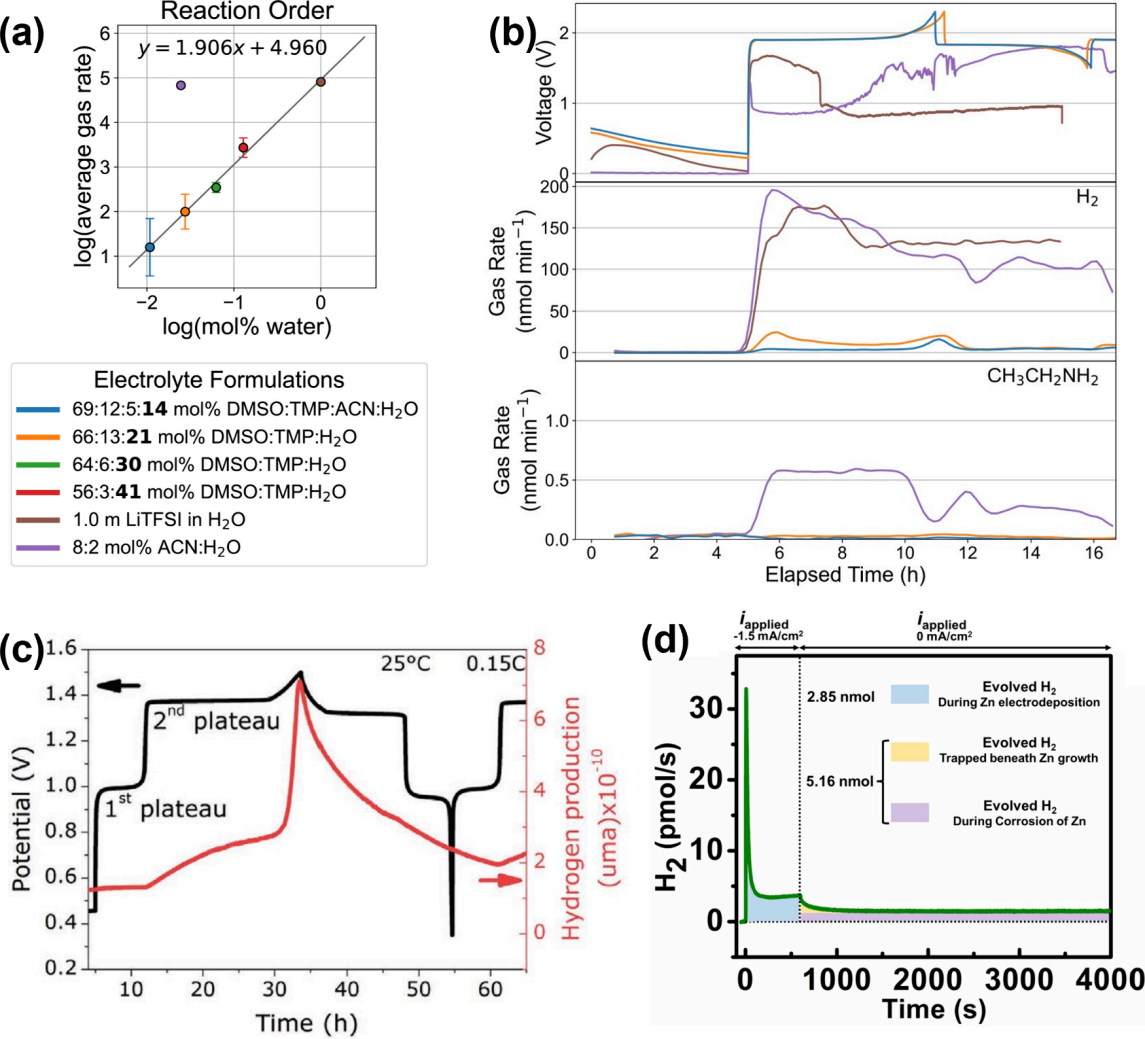
H_2_ evolution in aqueous batteries. (a) Logarithm-logarithm plot of the gas evolution rate and water concentration to determine any significant deviations from the reaction order trend line. (b) Voltage and gas evolution profiles of representative optimized aqueous electrolytes using the ODACell 2 platform. Adapted with permission under a Creative Commons CC-By 4.0 license from ref 26. Copyright 2025 Cell Press. (c) Mo_6_S_8_/LiFePO_4_ full-cell voltage profile and hydrogen evolution profile as functions of time at 0.15C. Reproduced with permission from ref 29. Copyright 2020 Wiley. (d) Quantification of evolved H_2_ gas as a function of time for aqueous Zn batteries. Reprinted with permission under a Creative Commons CC-By 4.0 license from ref 30. Copyright 2024 Wiley.

Droguet and co-workers scrutinized the effectiveness of the SEI formed in superconcentrated aqueous electrolytes, also known as “water-in-salt” electrolytes (WiSEs), in suppressing HER and self-discharge.^29^ Using an LiFePO_4_ | 20 M LiTFSI | Mo_6_S_8_ aqueous chemistry, the authors observed H_2_ evolution as soon as the Mo_6_S_8_ electrode reached its second lithiation voltage plateau at 1.4 V (Figure 2(c)). This suggests that any SEI formed was not able to completely prevent water reduction. Moreover, once the Mo_6_S_8_ electrode was fully lithiated, a much faster H_2_ evolution rate was observed toward the end of charge, as all electrons were consumed by the HER.

Apart from aqueous Li-ion batteries, aqueous Zn batteries are also receiving substantial attention owing to the high theoretical capacity (820 mAh g^–1^, 5854 mAh cm^–3^) and low redox potential (−0.76 V vs SHE) of Zn. However, the Zn anode intrinsically suffers from HER, impeding the commercialization of the aqueous chemistry. To quantify the number of electrons transferred to HER, Roy and co-workers electrochemically deposited 1.5 mA cm^–2^ of Zn on a Cu substrate for 600 s and monitored H_2_ evolution using *in situ* electrochemical mass spectrometry.^30^ As shown in Figure 2(d), the initial spike in H_2_ may stem from initial HER on the bare Cu substrate prior to massive Zn deposition. The total amount of H_2_ evolved during Zn plating was 2.84 nmol (up to 600 s). The faradaic efficiency for hydrogen evolution was 0.31%, while, by assuming no other electron transfer-driven side reactions, the remaining charge (99.69%) was used for Zn plating. When the current was stopped, the hydrogen evolution continued for thousands of seconds, which was ascribed to continuous Zn corrosion (Zn + 2H^*+*^ → Zn^2+^ + H_2_ *↑*).

### 3.2 O_2_

While OER has been less frequently focused on than HER for aqueous Li-ion batteries, the redox potential of certain positive electrode materials, such as spinel-type LiMn_2_O_4_ (ca. 4.0 and 4.2 V vs Li^+^/Li^0^, equivalent to ca. 1.0 and 1.2 V vs SHE), is close to that of the OER. Hou and co-workers conducted OEMS experiments on LiMn_2_O_4_ | 2 m LiTFSI | V_2_O_5_ aqueous cells with different N/P ratios.^31^ With an N/P ratio of 1:2 and an upper cutoff voltage of 1.8 V, as illustrated in Figure 3(a), signals at the ion current of *m*/*z* = 32, corresponding to oxygen evolution, were readily identified during charge of the aqueous full-cell. With an optimized N/P ratio of 1:2.5 (Figure 3(b)), neither H_2_ nor O_2_ was recorded. However, when the upper cutoff voltage was extended to 2 V, significant hydrogen evolution was observed at the same N/P ratio (Figure 3(c)). The results confirm the vital importance of balancing the electrode and optimizing cutoff conditions in order to minimize cell gassing for realistic cycling tests.

**Figure 3:**
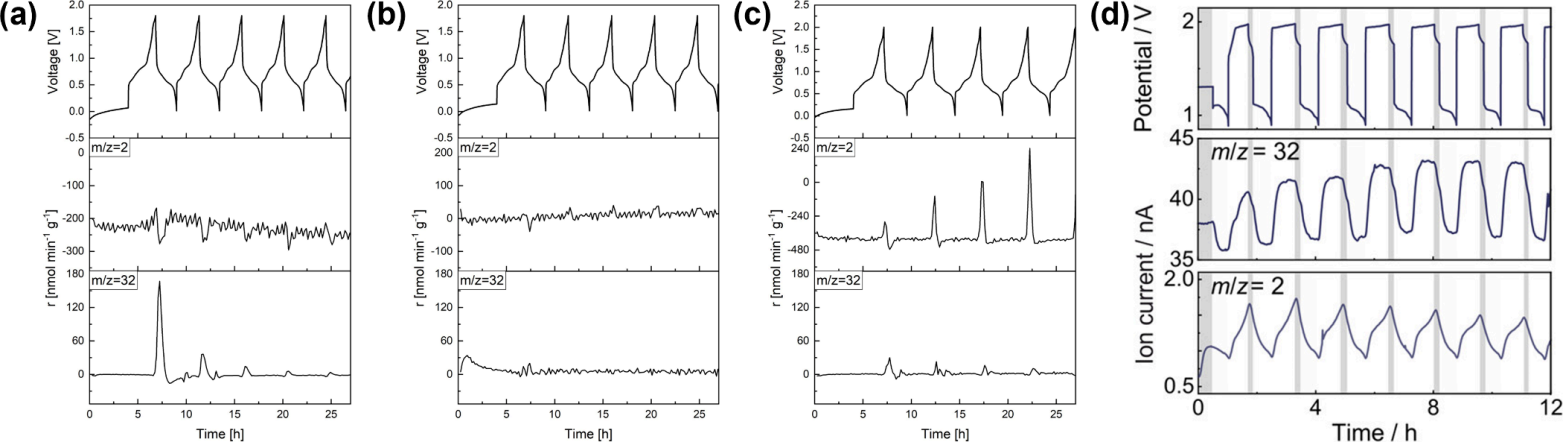
O_2_ evolution in aqueous batteries. H_2_ and O_2_ evolution of LiMn_2_O_4_/V_2_O_5_ aqueous full-cells with (a) an N/P ratio of 1:2 and an upper cutoff voltage of 1.8 V, (b) an N/P ratio of 1:2.5 and an upper cutoff voltage of 1.8 V, and (c) an N/P ratio of 1:2.5 and an upper cutoff voltage of 2 V. Adapted with permission under a Creative Commons CC-BY-NC 4.0 license from ref 31. Copyright 2024 Wiley. (d) H_2_ and O_2_ profiles of an aqueous Zn–O_2_ cell. Reprinted with permission under a Creative Commons CC-BY 4.0 license from ref 32. Copyright 2020 American Chemical Society.

Another highly relevant cell chemistry is aqueous metal oxygen batteries, in which molecular oxygen is directly involved in the reversible electrode redox reactions. For instance, Dongmo and co-workers applied DEMS analysis to study the gas evolution in aqueous Zn–O_2_ batteries with a Zn foil anode.^32^ In Figure 3(d), the authors identified repeated H_2_ evolution, which was attributed to Zn corrosion. Signals at the ion current of *m*/*z* = 32 were ascribed to oxygen evolution and consumption, which decreased during discharge $\left(\frac{1}{2}O_{2}+H_{2}O+2e^{-}\to 2{\mathrm{OH}}^{-}\right)$ and increased during charge $\left(2{\mathrm{OH}}^{-}\to \frac{1}{2}O_{2}+H_{2}O+2e^{-}\right)$ . The quantification of gaseous species is particularly relevant for mechanistic investigations of metal-gas batteries with both aqueous and nonaqueous electrolytes.

### 3.3 CO_2_

CO_2_ is known to be generated through carbon corrosion in aqueous fuel cells, electrolyzers, and supercapacitors. To explore if such reactions exist in aqueous batteries, Hou and co-workers applied OEMS in combination with element isotope labeling to investigate conductive carbon corrosion in a model LiMn_2_O_4_ | 2 m LiTFSI | V_2_O_5_ aqueous chemistry.^31^ ^13^C-enriched conductive carbon was purchased and used for electrode slurry coating. As shown in Figure 4(a), when neither of the electrodes was ^13^C-labeled, 99% of the CO_2_ detected was ^12^CO_2_, as the natural abundance of ^13^C is only 1.1%. However, when the conductive carbon of the LMO electrode was ^13^C-labeled (Figure 4(b)), substantial ^13^CO_2_ was detected at *m*/*z* = 45, confirming that carbon corrosion (C + 2H_2_O → CO_2_ + 4e^–^ + 4H^+^) exists in aqueous batteries as well. Interestingly, when the V_2_O_5_ negative electrode was prepared with ^13^C-labeled conductive carbon (Figure 4(c)), a new CO_2_ evolution peak was observed at the end of discharge, at which the electrode potential of V_2_O_5_ was high enough to trigger carbon corrosion of the negative electrode. The study demonstrates isotope-labeling as an effective strategy to discriminate the origin of gas evolution in aqueous batteries.

**Figure 4:**
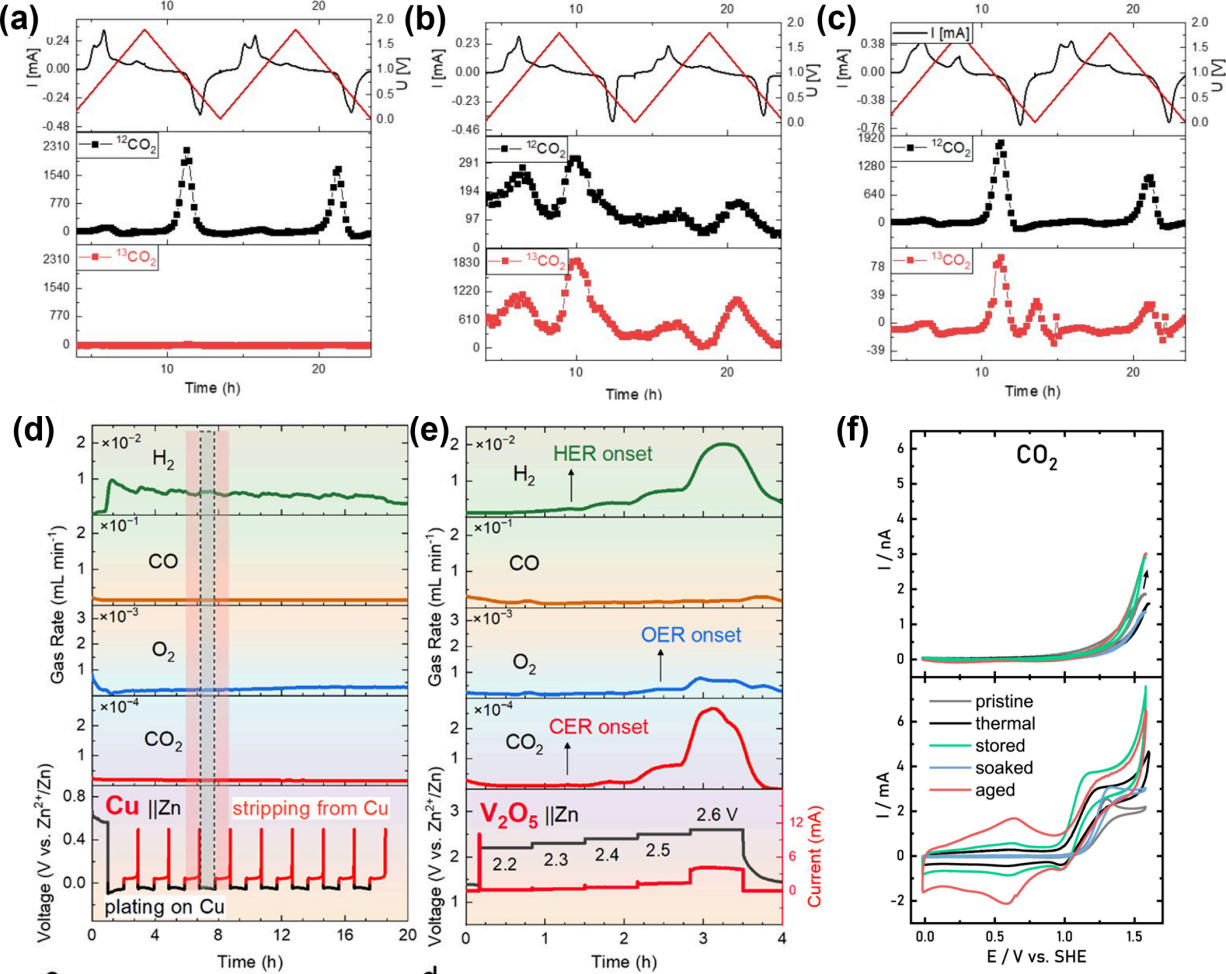
CO_2_ evolution in aqueous batteries. ^12^CO_2_ (*m*/*z* = 44) and ^13^CO_2_ (*m*/*z* = 45) signals of (a) normal LiMn_2_O_4_/V_2_O_5_ electrodes, (b) ^13^C-labeled LiMn_2_O_4_ and normal V_2_O_5_ electrodes, and (c) normal LiMn_2_O_4_ and ^13^C-labeled V_2_O_5_ electrodes. Reprinted with permission under a Creative Commons CC-BY-NC 4.0 license from ref 31. Copyright 2024 Wiley. DEMS analysis of H_2_, CO, O_2_, and CO_2_ in (d) Cu/Zn cell and (e) V_2_O_5_/Zn cells. Reproduced with permission from ref 34. Copyright 2024 American Chemical Society. (f) CV curves and CO_2_ evolution profiles of carbon felts for aqueous vanadium redox flow batteries. Reprinted with permission under a Creative Commons CC-BY-NC-ND 4.0 license from ref 35. Copyright 2020 Elsevier.

Carbon corrosion has also been observed by Wu and co-workers in aqueous Zn batteries.^33,34^ In a Cu || Zn cell (Figure 4(d)), H_2_ was identified as the main gaseous product, which primarily evolved during Zn plating onto the Cu substrate. However, in a V_2_O_5_ || Zn full-cell (Figure 4(e)), oxidative gases evolution was observed during a chronoamperometry test with 0.1 V voltage increment in each cycle (up to 2.6 V vs Zn^2+^/Zn^0^). O_2_ was ascribed to catalytic H_2_O oxidation, which occurred at 2.5 V. CO_2_ was identified in the medium-voltage of ca. 2.3 V, which, with the help of isotope-labeling, was confirmed to stem from conductive carbon oxidation.

Eifert and co-workers studied the CO_2_ evolution of pretreated carbon felt electrodes for vanadium redox flow batteries.^35^ Figure 4(f) shows the cyclic voltammograms and CO_2_ evolution profiles of carbon felts in a 2 M H_2_SO_4_ electrolyte containing 5 mM VOSO_4_ under flow conditions. A steep increase in current is observed around 0.9–1 V, which can be attributed to the oxidation of V^4+^. Massive CO_2_ evolution occurred mainly above 1.45 V, during which another increase in the current response is observed. It can be seen that thermal treatment can reduce the CO_2_ evolution rate, owing to the removal of volatile components. Meanwhile, the electrochemically aged and stored felts result in a significant increase in CO_2_, which can likely be attributed to the partial oxidation of the carbon surface.

### 3.4 H_2_S

OEMS has also been applied to study more complex systems with less common gaseous products. For example, Chao and co-workers conducted DEMS analysis to study gas evolution in sulfur aqueous batteries (SABs).^36,37^ As shown in Figure 5(a), by saturating the electrolyte with triflate anions (OTf^–^), the formation of H_2_S as a degradation product of the sulfur cathode was eliminated. Zhang and co-workers investigated the degradation mechanism of LiMn_2_O_4_ | 2 m LiTFSI | TiS_2_ aqueous full-cells.^38^ The electrochemical performance was assessed using a three-electrode Swagelok cell, while the gas evolution was analyzed by OEMS (Metrilytics AB, Sweden). A screenshot of the graphical user interface is illustrated in Figure 5(b), demonstrating the customizable real-time data collection from multiple OEMS cells. H_2_ is seen from Figure 5(c) as the major gaseous product, whose evolution is governed by the potential of the TiS_2_ electrode. In addition, signals at *m*/*z* = 34 and 64 were periodically detected, which can be attributed to the formation of H_2_S and trace S_8_. Although SO_2_ could also contribute to the ion current at *m*/*z* = 64, no signal at *m*/*z* = 48 was observed, excluding such a hypothesis. It is worth noting that the declining formation rate of H_2_S is accompanied by an increasing H_2_ evolution rate, suggesting that the two processes are highly correlated.

**Figure 5:**
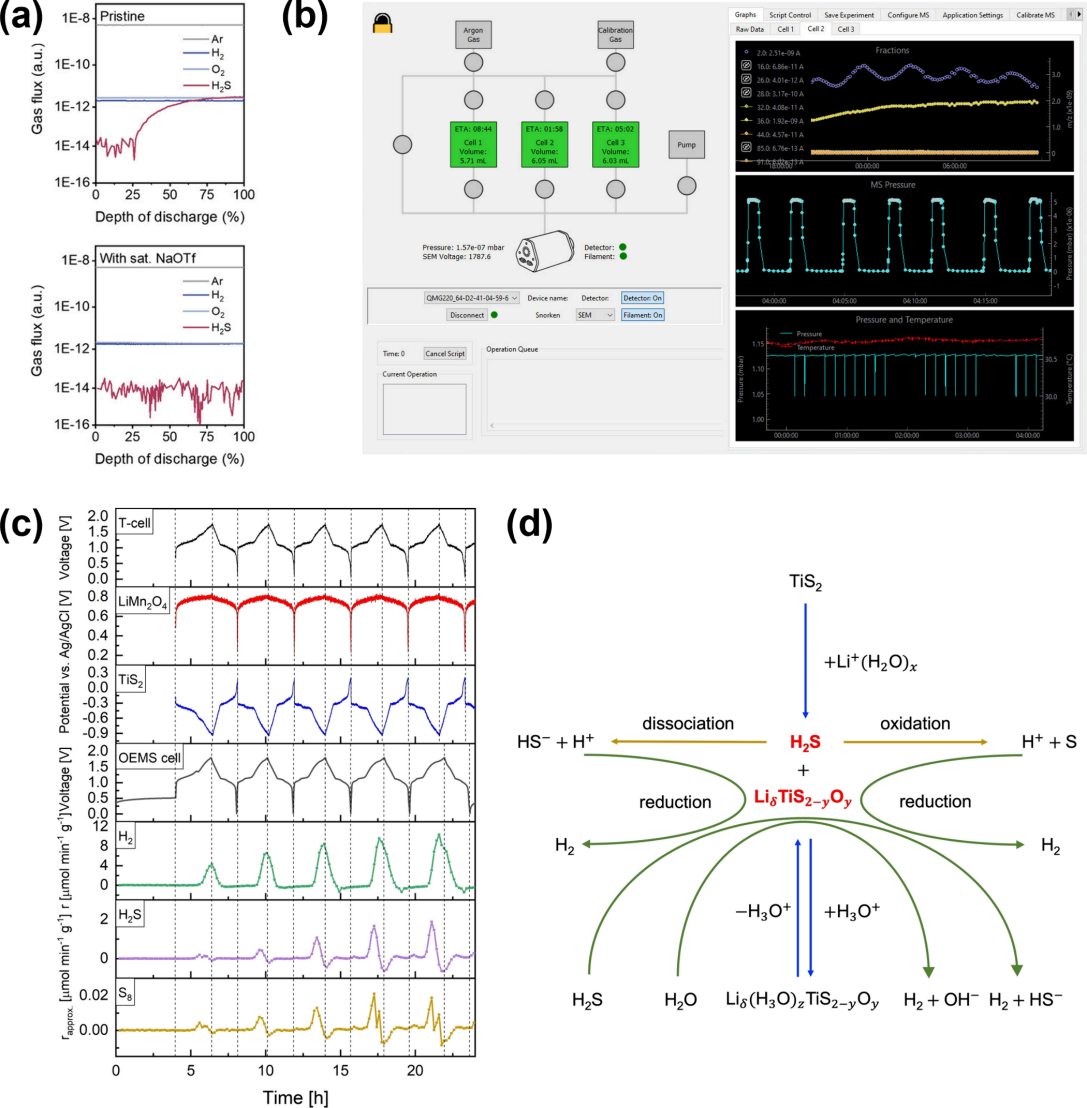
H_2_, H_2_S, and S_8_ evolution in aqueous batteries. (a) Gas evolution of sulfur aqueous batteries with 3 mol kg^–1^ and 9 mol kg^–1^ NaOTf electrolytes. Reproduced with permission from ref 36. Copyright 2025 Wiley. (b) Screenshot of the OEMS graphical user interface, demonstrating customizable real-time data collection from multiple OEMS cells. (c) Operando gas analysis coupled with three-electrode measurements for LiMn_2_O_4_/TiS_2_ aqueous full-cells. (d) Schematic diagram showing the reaction mechanism of TiS_2_ in aqueous electrolytes. Reprinted with permission under a Creative Commons CC-BY-NC 4.0 license from ref 38. Copyright 2022 Wiley.

Based on these observations, the authors proposed a degradation mechanism for TiS_2_ in aqueous electrolytes, which is summarized in Figure 5(d). Specifically, TiS_2_ partially hydrolyzes in water, releasing H_2_S gas. The latter can either dissociate in water, forming HS^–^ and H^+^, or be oxidized to form H^+^ and S_8_. The protons thus generated can be electrocatalytically reduced to form H_2_. In addition, either H_2_O or H_2_S may be directly reduced on the electrode surface to form H_2_. The partially hydrolyzed bulk phase will thereafter uptake and release water following a reversible hydration mechanism involving water cointercalation.^39^

## 4 Discussion and Future Perspectives

### 4.1 Comparison of Gas Analysis Techniques

Various gas analysis techniques have been adopted to study gas evolution in both aqueous and nonaqueous batteries. Table 1 lists several representative methods and compares their respective properties. OEMS stands out with its *operando* nature, being able to identify gas composition and quantify concentration.^38,40,41^ However, the technique requires a cumbersome mass spectrometer and accessories (pump, gas tubing, etc.) attached to the battery cell, which requires a high initial investment cost and takes up a large footprint. Several technical challenges associated with gas analysis in aqueous batteries will be further elaborated in Section 4.3.

**Table 1:** Comparison of Gas Analysis Techniques

**Technique**	***Operando*** **/** ***in situ***	**Composition**	**Concentration**	**Investment cost**	**Footprint**	**References**
**OEMS**	Yes	Yes	Yes	High	Large	^38^,^40^,^41^
**GC**(-MS)	Sometimes	Yes	Yes	High	Large	^28^,^42^,^43^
**Archimedes principle**	Yes	No	Only volume	Low	Small	^44^
**Pressure sensor**	Yes	No	Only pressure	Low	Small	^29^,^45^
**Optical fiber**	Yes	Specific	In ppm/ppb	Medium	Medium	^46^
**Ultrasound**	Yes	No	Qualitative	Medium	Medium	^47–49^
**Acoustic emission**	Yes	No	Qualitative	Low	Small	^25^,^50^

GC (often coupled with MS) is a commercially available analytical tool for gas quantification. It has been traditionally regarded as an *ex situ* method, but recent studies have also demonstrated its *operando* capability.^28,42,43^ Nevertheless, considering the residual time that gas needs to pass through the GC column, its time resolution is much lower than that of the OEMS.

An apparatus monitoring the volume change of Li-ion pouch-cells by Archimedes’ principle was reported by Dahn’s group.^44^ A hanging pouch-cell was submerged in silicone oil while the other end of the wire was connected to a balance. The change in buoyant force can be correlated to the volume change of the cell. Though simple and elegant, this method cannot identify the gas composition and concentration.

The gas evolution can also be quantified following the pressure change of the cell. Lepoivre and co-workers integrated a pressure sensor into a Swagelok-type cell for Li–O_2_ batteries.^45^ While the setup takes up a small footprint and is economically budgeted, similar to the previous case, no chemical information is given.

Optical fiber sensing has shown promising progress in studying gas evolution in batteries. As a proof-of-concept study, Zheng and co-workers employed hollow-core fiber photothermal spectroscopy (HCF-PTS) sensors to quantify C_2_H_4_ and CO_2_ evolution in classic LiFePO_4_ || graphite full-cells with a carbonate electrolyte.^46^ However, a sophisticated optical interrogation system needs to be constructed to perform the experiment, and the absorption line of the targeting gas needs to be looked up from databases like HITRAN^51^ in advance.

Last but not least, acoustic sensing also provides complementary information for battery gassing. For instance, ultrasound imaging was incorporated to study electrolyte wetting and gas evolution in pouch-cells with high spatial resolution.^47–49^ Acoustic emission sensing was also demonstrated to semiquantitatively correlate with the HER in aqueous Li-ion batteries.^25^ Unfortunately, acoustic methods do not reveal the chemical nature of the evolving gas.

### 4.2 Assessment of Cell Components

OEMS has proven to be a versatile *operando* technique for studying aqueous electrochemical systems. The gassing behavior of materials with dual energy storage–conversion properties is particularly interesting. For instance, the aforementioned transition metal dichalcogenide highlights how an electrode material for aqueous batteries can simultaneously electrocatalyze water splitting and produce H_2_.^38^ Similarly, Li-rich layered oxides undergoing anionic redox processes also play an integral role as electrocatalysts for OER.^52–54^ OEMS offers direct and quantitative evidence of gas evolution, shedding light on the design of advanced materials for batteries and electrocatalysis.

The commercialization of modern aqueous batteries has been impeded by the unstable nature of the SEI layer formed in aqueous electrolytes. Droguet and co-workers confirmed that the solubility of a conformal LiF coating on Li metal can be drastically reduced in highly concentrated aqueous electrolytes, but the as-formed LiF-coating layer cannot self-passivate and will always allow water to reach the anode material.^55^ Instead, electrolyte additives and/or cosolvents might be more feasible, assuming that they will preferentially degrade and form a stable SEI layer *in situ*; however, this assumption needs to be validated by OEMS.

In nonaqueous batteries, the formation of ethylene is characteristic of ethylene carbonate reduction and SEI formation. Drawing an analogy, one may apply OEMS to look for signature gaseous decomposition products of film-forming additives and assess the resulting H_2_ amounts in aqueous batteries. It is worth mentioning that externally supplied CO_2_ has been proposed to participate in the SEI formation of aqueous Li-ion batteries, resulting in a Li_2_CO_3_-rich SEI layer for relatively dilute aqueous electrolytes (5 m).^56^ As OEMS can purge the internal environment of battery cells with different gases, the technique can be highly beneficial to decipher the SEI formation mechanism with gaseous additives as well.

OEMS can also contribute to the endeavor of developing highly efficient metal anodes (Zn, Fe, Mn, Al, etc.) for next-generation aqueous batteries. Compared to conventional intercalation chemistry, metal anodes follow a distinct plating–stripping mechanism, enabling multielectron transfer and an enlarged cell voltage. However, these metals are typically outside the water ESW; the continuous hydrogen evolution, metal corrosion, and uncontrolled dendrite growth significantly hamper their commercial application. Applying OEMS offers transformative opportunities to address these critical challenges. Apart from the electrolyte engineering approach mentioned earlier, OEMS can also assess the gassing behaviors of electrode foil modification strategies, such as 3D host architectures, alloying anodes, surface coating, and initially anode-free configurations. However, it should be noted that metal anodes have relatively less surface area than porous composite electrodes. Consequently, the design and optimization for OEMS cells and systems should be further addressed.

### 4.3 Technical Design Aspects

The technical design of OEMS for aqueous batteries revolves around three key aspects: sensitivity, accuracy, and productivity. As the MS is a well-established technique with a sensitivity down to the parts-per-million level, the detection limit is subject to the airtightness of the OEMS cell and its interface with the MS. Therefore, it is crucial to design the system using high-fidelity gas connections. For example, Lundström and Berg constructed the gas sampling system with Swagelok VCR connectors, sealed with silver-plated gaskets to minimize gas leakage.^16^

Another source contributing to OEMS background signals is the use of volatile solvents in conventional nonaqueous batteries. For instance, linear carbonates, such as dimethyl carbonate and diethyl carbonate, contribute to overlapping signals around ion currents of *m*/*z* = 25–30, which limits the differentiation of several common gases, including C_*x*_H_*y*_, CO, and N_2_.^57^ Fortunately, as water is the sole solvent for aqueous batteries, its mass spectrum does not interfere with other gases. Applying a cold trap before the gas enters the MS may further lower the background signal.

In the context of aqueous batteries, particularly full-cells, controlling electrode potential is of vital importance to the system’s performance and longevity. In Li half cells, Li metal can be regarded as both the counter and reference electrode. However, using a metal foil as the reference electrode in aqueous cells is less practical, owing to the challenges of continuous HER, metal corrosion, and surface passivation. Therefore, designing an adapted OEMS cell for aqueous batteries with an embedded reference electrode, preferably commercially available ones, such as a saturated calomel electrode or an Ag/AgCl electrode, is highly relevant. Nevertheless, introducing a new component to the existing cell design requires several iterations to minimize additional leakage challenges, which should be carefully considered.

With the advancement of high-throughput testing and lab automation, numerous routine procedures can be carried out autonomously, which is intrinsically more accurate, reproducible, and efficient. For example, Jia and co-workers designed a high-throughput workflow for materials synthesis, characterization, and electrochemical testing for Na-ion batteries.^58^ Yik and co-workers reported a closed-loop workflow for automating high-throughput electrolyte mixing, coin cell assembly, and electrochemical testing for aqueous batteries.^26,59^ It is worth mentioning that high-throughput mass spectrometry has accelerated the identification of active chemicals in early drug discovery pipelines within the pharmaceutical industry.^60^ Learning from the experience, it is technically possible to automate the assembly and testing of OEMS cells for (non)­aqueous batteries. The massive data set thus generated can be analyzed with machine-learning algorithms. For example, MassQL, proposed by Damiani and co-workers, allows users to write structured queries that search MS data sets for characteristic ion signatures of fragmentation patterns.^61^ This can be highly relevant for screening electrolyte additives for hydrogen suppression and discovering unknown gas-phase degradation products across high-throughput data sets.

## 5 Summary

The past three decades have witnessed the evolution of online electrochemical mass spectrometry as a highly valuable technique for quantifying side reactions across a wide range of battery chemistries, with particular relevance to aqueous systems. The technique enables the *operando* identification of volatile species generated within the cell. Owing to the narrow ESW of water, hydrogen and oxygen evolutions have been the primary focus so far, while a growing trend has been observed in utilizing the technique to study less common yet equally important gases, such as CO_2_ and H_2_S. This Perspective has outlined both the practical applications of OEMS, such as the in-depth analysis of electrodes, electrolytes, and interphases, as well as future technical improvements needed to further optimize the technique for aqueous battery systems. With companies like Metrilytics AB (Sweden), Spectro Inlets (Denmark), LIQUIDLOOP GmbH (Germany), Linglu Instruments (China), and Hiden Analytical (UK) offering services and commercializing the technique, the author truly expects OEMS to evolve into a more widely adopted methodology, gaining traction within the rapidly expanding aqueous battery research community and supporting the development of safer, more efficient, and longer-lasting energy storage technologies.

## References

[1] Zhang L., Zhang C., Berg E. J. (2025). Mastering Proton Activities in Aqueous
Batteries. Adv. Mater..

[2] Ko S., Nishimura S. I., Takenaka N., Kitada A., Yamada A. (2025). Practical
Issues toward High-Voltage Aqueous Rechargeable Batteries. Chem. Soc. Rev..

[3] Suo L., Borodin O., Gao T., Olguin M., Ho J., Fan X., Luo C., Wang C., Xu K. (2015). “Water-in-Salt”
Electrolyte Enables High-Voltage Aqueous Lithium-Ion Chemistries. Science.

[4] Yamada Y., Usui K., Sodeyama K., Ko S., Tateyama Y., Yamada A. (2016). Hydrate-Melt Electrolytes for High-Energy-Density
Aqueous
Batteries. Nat. Energy.

[5] Xie J., Liang Z., Lu Y. C. (2020). Molecular
Crowding Electrolytes for
High-Voltage Aqueous Batteries. Nat. Mater..

[6] Hao J., Yuan L., Ye C., Chao D., Davey K., Guo Z., Qiao S. (2021). Boosting Zinc
Electrode Reversibility in Aqueous Electrolytes
by Using Low-Cost Antisolvents. Angew. Chem..

[7] Sui Y., Ji X. (2021). Anticatalytic
Strategies to Suppress Water Electrolysis in Aqueous
Batteries. Chem. Rev..

[8] Li H., Cao M., Wang R., Xiong P., Liu Y., Zhang L., Zhang L., Zhang L., Chao D., Zhang C. (2025). Design Strategy
for Small-Molecule Organic Cathodes: Regulated Active Groups Enable
High Capacity and Voltage in Aqueous and Seawater Aluminium Ion. Batteries. Angew. Chemie Int. Ed..

[9] Zhang W., Liu Y., Luo X., Wang R., Zhou K., Yuan L., Li F., Li H., Zhang L., Zhang C. (2025). Multi-Solvent Synergy
Strategy Unlocks Anti-Corrosion and High Reversibility of Zinc Anodes:
Paving the Way for Robust and Temperature-Resilient Zinc-Iodine Batteries. Adv. Funct. Mater..

[10] Liu L., Zhang L., Liu Y., Zhang S., Wang R., Li F., Li H., Hao J., Zhang C., Guo Z. (2025). Enhanced Redox
Kinetics of Aqueous I–/I2/I+ Conversion Chemistry in Hydrated
Eutectic Electrolyte Over a Wide Temperature Range. Adv. Energy Mater..

[11] Wang, Q. ; Zhou, W. ; Zhang, Y. ; Jin, H. ; Li, X. ; Zhang, T. ; Wang, B. ; Zhao, R. ; Zhang, J. ; Li, W. ; Qiao, Y. ; Jia, C. ; Zhao, D. ; Chao, D. Rescue of Dead MnO2 for Stable Electrolytic Zn–Mn Redox-Flow Battery: A Metric of Mediated and Catalytic Kinetics. Natl. Sci. Rev. 2024, 11 (8). 10.1093/nsr/nwae230. PMC11312367 39131921

[12] Zheng T., Muneeswara M., Bao H., Huang J., Zhang L., Hall D. S., Boles S. T., Jin W. (2024). Gas Evolution
in Li-Ion
Rechargeable Batteries: A Review on Operando Sensing Technologies,
Gassing Mechanisms, and Emerging Trends. ChemElectroChem..

[13] Kim, S. ; Kim, H. S. ; Kim, B. ; Kim, Y. J. ; Jung, J. W. ; Ryu, W. H. In Situ Gas Analysis by Differential Electrochemical Mass Spectrometry for Advanced Rechargeable Batteries: A Review. Adv. Energy Mater. 2023, 13 (39). 10.1002/aenm.202301983.

[14] Tang G., Zhang J., Ma S., Li J., Peng Z., Chen W. (2025). Unveiling Gas Production in Rechargeable Batteries via in Situ Differential
Electrochemical Mass Spectrometry. Chem. Soc.
Rev..

[15] Novák P., Goers D., Hardwick L., Holzapfel M., Scheifele W., Ufheil J., Würsig A. (2005). Advanced in
Situ Characterization Methods Applied to Carbonaceous Materials. J. Power Sources.

[16] Lundström R., Berg E. J. (2021). Design and Validation
of an Online Partial and Total
Pressure Measurement System for Li-Ion Cells. J. Power Sources.

[17] McCloskey B. D., Bethune D. S., Shelby R. M., Girishkumar G., Luntz A. C. (2011). Solvents Critical Role in Nonaqueous
Lithium-Oxygen
Battery Electrochemistry. J. Phys. Chem. Lett..

[18] Tsiouvaras N., Meini S., Buchberger I., Gasteiger H. A. (2013). A Novel
On-Line Mass Spectrometer Design for the Study of Multiple Charging
Cycles of a Li-O2 Battery. J. Electrochem. Soc..

[19] Berkes B. B., Jozwiuk A., Sommer H., Brezesinski T., Janek J. (2015). Simultaneous Acquisition of Differential Electrochemical Mass Spectrometry
and Infrared Spectroscopy Data for in Situ Characterization of Gas
Evolution Reactions in Lithium-Ion Batteries. Electrochem. commun..

[20] Sim R., Langdon J., Manthiram A. (2023). Design of an Online Electrochemical
Mass Spectrometry System to Study Gas Evolution from Cells with Lean
and Volatile Electrolytes. Small Methods.

[21] Kim Y., Jung J., Yu H., Kim G. T., Jeong D., Bresser D., Kang S. J., Kim Y., Passerini S. (2020). Sodium Biphenyl
as Anolyte for Sodium–Seawater Batteries. Adv. Funct. Mater..

[22] Rana A., Roy K., Heil J. N., Nguyen J. H., Renault C., Tackett B. M., Dick J. E. (2024). Realizing
the Kinetic Origin of Hydrogen Evolution
for Aqueous Zinc Metal Batteries. Adv. Energy
Mater..

[23] Zhang J., Wang Y., Zhao Z., Li P., Tang G., Chen W., Peng Z. (2024). Unveiling the Descriptor of Parasitic
Reactions of Zinc Anode: A Comparative Study of Trace Pyridinesulfonic
Acid-Based Additives in Aqueous Electrolyte. Adv. Energy Mater..

[24] Luo X., Wang R., Zhang L., Liu Z., Li H., Mao J., Zhang S., Hao J., Zhou T., Zhang C. (2024). Air-Stable
and Low-Cost High-Voltage Hydrated Eutectic Electrolyte for High-Performance
Aqueous Aluminum-Ion Rechargeable Battery with Wide-Temperature Range. ACS Nano.

[25] Espinoza
Ramos I., Guo Z., Clulow R., Su B., Zhao Q., Holm Gjørup F., Ahlberg Tidblad A., Zhang L. (2025). Unveiling Chemomechanical Degradation in Aqueous Batteries with Online
Acoustic Emission Sensing. Mater. Today Energy.

[26] Yik J. T., Hvarfner C., Sjölund J., Berg E. J., Zhang L. (2025). Accelerating
Aqueous Electrolyte Design with Automated Full-Cell Battery Experimentation
and Bayesian Optimization. Cell Reports Phys.
Sci..

[27] Xia R., Tian D., Kattel S., Hasa B., Shin H., Ma X., Chen J. G., Jiao F. (2021). Electrochemical Reduction of Acetonitrile
to Ethylamine. Nat. Commun..

[28] Paillot M., Caër S., Le, Gauthier M. (2025). Is the Amount
of Water the Most Important
Parameter in Concentrated Aqueous Electrolytes? The Case of Aqueous
Magnesium Cells. ACS Electrochem..

[29] Droguet L., Grimaud A., Fontaine O., Tarascon J. M. (2020). Water-in-Salt Electrolyte
(WiSE) for Aqueous Batteries: A Long Way to Practicality. Adv. Energy Mater..

[30] Roy K., Rana A., Heil J. N., Tackett B. M., Dick J. E. (2024). For Zinc
Metal Batteries, How Many Electrons Go to Hydrogen Evolution? An Electrochemical
Mass Spectrometry Study. Angew. Chemie - Int.
Ed..

[31] Hou X., Zhang L., Gogoi N., Edström K., Berg E. J. (2024). Interfacial Chemistry in Aqueous Lithium-Ion Batteries:
A Case Study of V2O5 in Dilute Aqueous Electrolytes. Small.

[32] Dongmo S., Stock D., Alexander Kreissl J.
J., Groß M., Weixler S., Hagen M., Miyazaki K., Abe T., Schröder D. (2020). Implications of Testing a Zinc-Oxygen Battery with
Zinc Foil Anode Revealed by Operando Gas Analysis. ACS Omega.

[33] Wu Z., Li Y., Amardeep A., Shao Y., Zhang Y., Zou J., Wang L., Xu J., Kasprzak D., Hansen E. J., Liu J. (2024). Unveiling the Mysteries: Acetonitrile’s Dance with Weakly-Solvating
Electrolytes in Shaping Gas Evolution and Electrochemical Performance
of Zinc-Ion. Batteries. Angew. Chemie - Int.
Ed..

[34] Wu Z., Shao Y., Hansen E. J., Tao L., Mir R. A., Kasprzak D., Liu J. (2024). Carbon Dioxide Evolution in Aqueous
Zinc Metal Batteries. ACS Appl. Mater. Interfaces.

[35] Eifert L., Jusys Z., Behm R. J., Zeis R. (2020). Side Reactions and
Stability of Pre-Treated Carbon Felt Electrodes for Vanadium Redox
Flow Batteries: A DEMS Study. Carbon N. Y..

[36] Yu X., Feng Y., Tian J., Liu X., Wang B., Zhang Y., Zhang T., Li G., Li X., Jin H., Zhou W., Li W., Zeng Z., Li L., Zhao D., Chao D. (2025). Unveil the Failure of Alkali Ion-Sulfur
Aqueous Batteries: Resolving Water Migration by Coordination. Regulation. Angew. Chemie - Int. Ed..

[37] Li X., Zhang T., Li G., Wang B., Jin H., Zhang Y., Liu X., Feng Y., Wang Y., Zhou W., Zhao J., Li W., Fan H., Zhao D., Chao D. (2025). A Mn2+-S Redox Electrochemistry
for
Energetic Aqueous Manganese Ion Battery. Joule.

[38] Zhang L., Hou X., Edström K., Berg E. J. (2022). Reactivity of TiS2
Anode towards Electrolytes in Aqueous Lithium-Ion Batteries. Batter. Supercaps.

[39] Zhang L., Kühling F., Mattsson A.-M., Knijff L., Hou X., Ek G., Dufils T., Gjørup F. H., Kantor I., Zhang C., Brant W. R., Edström K., Berg E. J. (2024). Reversible Hydration
Enabling High-Rate Aqueous Li-Ion Batteries. ACS Energy Lett..

[40] Wang Y., Wang T., Dong D., Xie J., Guan Y., Huang Y., Fan J., Lu Y. C. (2022). Enabling
High-Energy-Density
Aqueous Batteries with Hydrogen Bond-Anchored Electrolytes. Matter.

[41] Chen Q., Hao J., Zhang S., Tian Z., Davey K., Qiao S. Z. (2024). High-Reversibility
Sulfur Anode for Advanced Aqueous Battery. Adv.
Mater..

[42] Zhang H., Wu X., Li Z., Zou Y., Wang J., Yu X., Chen J., Xue J., Zhang B., Tian J. H., Hong Y.-h., Qiao Y., Sun S.-G. (2024). Full-Dimensional
Analysis of Gaseous Products Within Li-Ion Batteries by On-Line GC-BID/MS. Adv. Energy Mater..

[43] Gachot G., Ribière P., Mathiron D., Grugeon S., Armand M., Leriche J. B., Pilard S., Laruelle S. (2011). Gas Chromatography/Mass
Spectrometry as a Suitable Tool for the Li-Ion Battery Electrolyte
Degradation Mechanisms Study. Anal. Chem..

[44] Aiken C. P., Xia J., Wang D. Y., Stevens D. A., Trussler S., Dahn J. R. (2014). An Apparatus
for the Study of In Situ Gas Evolution in Li-Ion Pouch Cells. J. Electrochem. Soc..

[45] Lepoivre F., Grimaud A., Larcher D., Tarascon J.-M. (2016). Long-Time and Reliable
Gas Monitoring in Li-O2 Batteries via a Swagelok Derived Electrochemical
Cell. J. Electrochem. Soc..

[46] ZHENG T., Bao H., Chen F., Wu J., Zhao P., Ho H. L., Gao S., Wang Y., Huang J., Zhang L., Boles S., Jin W. (2025). Operando Monitoring
of Gassing Dynamics in Lithium-Ion Batteries
with Optical Fiber Photothermal Spectroscopy. Energy Environ. Sci..

[47] Xu W., Yang Y., Shi F., Li L., Wen F., Chen Q. (2023). Ultrasonic Phased Array Imaging of
Gas Evolution in a Lithium-Ion
Battery. Cell Reports Phys. Sci..

[48] Deng Z., Huang Z., Shen Y., Huang Y., Ding H., Luscombe A., Johnson M., Harlow J. E., Gauthier R., Dahn J. R. (2020). Ultrasonic Scanning
to Observe Wetting and “Unwetting”
in Li-Ion Pouch Cells. Joule.

[49] Huo H., Huang K., Luo W., Meng J., Zhou L., Deng Z., Wen J., Dai Y., Huang Z., Shen Y., Guo X., Ji X., Huang Y. (2022). Evaluating
Interfacial Stability in Solid-State Pouch Cells via Ultrasonic Imaging. ACS Energy Lett..

[50] Samantaray Y., Cogswell D. A., Cohen A. E., Bazant M. Z. (2025). Electrochemically
Resolved Acoustic Emissions from Li-Ion Batteries. Joule.

[51] Gordon I. E., Rothman L. S., Hargreaves R. J., Hashemi R., Karlovets E. V., Skinner F. M., Conway E. K., Hill C., Kochanov R. V., Tan Y., Wcisło P., Finenko A. A., Nelson K., Bernath P. F., Birk M., Boudon V., Campargue A., Chance K. V., Coustenis A., Drouin B. J., Flaud J. M., Gamache R. R., Hodges J. T., Jacquemart D., Mlawer E. J., Nikitin A. V., Perevalov V. I., Rotger M., Tennyson J., Toon G. C., Tran H., Tyuterev V. G., Adkins E. M., Baker A., Barbe A., Canè E., Császár A. G., Dudaryonok A., Egorov O., Fleisher A. J., Fleurbaey H., Foltynowicz A., Furtenbacher T., Harrison J. J., Hartmann J. M., Horneman V. M., Huang X., Karman T., Karns J., Kassi S., Kleiner I., Kofman V., Kwabia-Tchana F., Lavrentieva N. N., Lee T. J., Long D. A., Lukashevskaya A. A., Lyulin O. M., Makhnev V. Y., Matt W., Massie S. T., Melosso M., Mikhailenko S. N., Mondelain D., Müller H. S. P., Naumenko O. V., Perrin A., Polyansky O. L., Raddaoui E., Raston P. L., Reed Z. D., Rey M., Richard C., Tóbiás R., Sadiek I., Schwenke D. W., Starikova E., Sung K., Tamassia F., Tashkun S. A., Vander Auwera J., Vasilenko I. A., Vigasin A. A., Villanueva G. L., Vispoel B., Wagner G., Yachmenev A., Yurchenko S. N. (2022). The HITRAN2020 Molecular Spectroscopic
Database. J. Quant. Spectrosc. Radiat. Transfer.

[52] Grimaud A., Hong W. T., Shao-Horn Y., Tarascon J. M. (2016). Anionic Redox Processes
for Electrochemical Devices. Nat. Mater..

[53] Yang C., Rousse G., Louise Svane K., Pearce P. E., Abakumov A. M., Deschamps M., Cibin G., Chadwick A. V., Dalla
Corte D. A., Anton Hansen H., Vegge T., Tarascon J. M., Grimaud A. (2020). Cation Insertion to Break the Activity/Stability Relationship
for Highly Active Oxygen Evolution Reaction Catalyst. Nat. Commun..

[54] Grimaud A., Diaz-Morales O., Han B., Hong W. T., Lee Y. L., Giordano L., Stoerzinger K. A., Koper M. T. M., Shao-Horn Y. (2017). Activating
Lattice Oxygen Redox Reactions in Metal Oxides to Catalyse Oxygen
Evolution. Nat. Chem..

[55] Droguet L., Hobold G. M., Lagadec M. F., Guo R., Lethien C., Hallot M., Fontaine O., Tarascon J. M., Gallant B. M., Grimaud A. (2021). Can an Inorganic Coating Serve as
Stable SEI for Aqueous
Superconcentrated Electrolytes. ACS Energy Lett..

[56] Yue J., Zhang J., Tong Y., Chen M., Liu L., Jiang L., Lv T., Hu Y. S., Li H., Huang X., Gu L., Feng G., Xu K., Suo L., Chen L. (2021). Aqueous Interphase
Formed by CO2 Brings Electrolytes
Back to Salt-in-Water Regime. Nat. Chem..

[57] Zhang L., Tsolakidou C., Mariyappan S., Tarascon J. M., Trabesinger S. (2021). Unraveling
Gas Evolution in Sodium Batteries by Online Electrochemical Mass Spectrometry. Energy Storage Mater..

[58] Jia S., Abdolhosseini M., Saglio L., Li Y., Kamel M., Lavertu J. D., Bazylevych S., Saïbi V., Cabelguen P. E., Kumakura S., McCalla E. (2025). High-Throughput
Study
Examining the Wide Benefit of Li Substitution in Oxide Cathodes for
Na-Ion Batteries. Electrochim. Acta.

[59] Yik J. T., Zhang L., Sjölund J., Hou X., Svensson P. H., Edström K., Berg E. J. (2023). Automated Electrolyte
Formulation
and Coin Cell Assembly for High-Throughput Lithium-Ion Battery Research. Digit. Discovery.

[60] Dueñas M. E., Peltier-Heap R. E., Leveridge M., Annan R. S., Büttner F. H., Trost M. (2023). Advances in High-throughput Mass Spectrometry in Drug Discovery. EMBO Mol. Med..

[61] Damiani T., Jarmusch A. K., Aron A. T., Petras D., Phelan V. V., Zhao H. N., Bittremieux W., Acharya D. D., Ahmed M. M. A., Bauermeister A., Bertin M. J., Boudreau P. D., Borges R. M., Bowen B. P., Brown C. J., Chagas F. O., Clevenger K. D., Correia M. S. P., Crandall W. J., Crüsemann M., Fahy E., Fiehn O., Garg N., Gerwick W. H., Gilbert J. R., Globisch D., Gomes P. W. P., Heuckeroth S., James C. A., Jarmusch S. A., Kakhkhorov S. A., Kang K., Bin, Kessler N., Kersten R. D., Kim H., Kirk R. D., Kohlbacher O., Kontou E. E., Liu K., Lizama-Chamu I., Luu G. T., Luzzatto Knaan T., Mannochio-Russo H., Marty M. T., Matsuzawa Y., McAvoy A. C., McCall L. I., Mohamed O. G., Nahor O., Neuweger H., Niedermeyer T. H. J., Nishida K., Northen T. R., Overdahl K. E., Rainer J., Reher R., Rodriguez E., Sachsenberg T. T., Sanchez L. M., Schmid R., Stevens C., Subramaniam S., Tian Z., Tripathi A., Tsugawa H., van der Hooft J. J. J., Vicini A., Walter A., Weber T., Xiong Q., Xu T., Pluskal T., Dorrestein P. C., Wang M. (2025). A Universal Language for Finding Mass Spectrometry Data Patterns. Nat. Methods.

